# The neutrophil–lymphocyte ratio as a risk factor for all-cause mortality among individuals with resolved HBV infection: evidence from the NHANES 1999–2018

**DOI:** 10.3389/fpubh.2024.1493439

**Published:** 2025-01-15

**Authors:** Chen Qiu, Chaojie Yu, Lanlan Yang, Siqi Liu, Qian Zhang, Shengnan Jia, Wenrui Wang, Zhenjing Jin, Dongdong Yu

**Affiliations:** ^1^Digestive Disease Center, Department of Hepatopancreatobiliary Medicine, The Second Hospital, Jilin University, Changchun, Jilin, China; ^2^Second Clinical Medical College, Guangzhou University of Chinese Medicine, Guangzhou, China

**Keywords:** neutrophil-lymphocyte ratio, resolved HBV infection, all-cause mortality, diabetes, NHANES, HBV

## Abstract

**Background:**

Inflammation is a critical component in the process of resolved hepatitis B virus (HBV) infection. The neutrophil-to-lymphocyte ratio (NLR) serves as a sensitive indicator of systemic inflammation and immune activation. Our study aimed to investigate the correlation between elevated NLR levels and the risk of all-cause mortality in patients with resolved HBV infection. Additionally, we evaluated the potential mediating effect of diabetes mellitus (DM) on this correlation.

**Methods:**

Our study enrolled 1,146 adult patients with resolved HBV infection from the National Health and Nutrition Examination Survey (NHANES) between 1999 and 2018. We utilized the Restricted Cubic Splines (RCS) and Maximum Selection Rank Statistical Method (MSRSM) to analyze the relationship between the NLR and the risk of all-cause mortality. The impact of NLR was evaluated using a weighted multivariate Cox regression model, and the model’s predictive accuracy was assessed using time-dependent Receiver Operating Characteristic (ROC) curves. An intermediary analysis was conducted to explore the potential influence of DM on the observed relationship.

**Results:**

During follow-up period of 103.54 ± 4.90 months, we recorded 207 deaths among the study participants. The analysis using the RCS method revealed a significant positive correlation between the NLR and the risk of all-cause mortality. Those with elevated NLR levels faced a substantially higher mortality risk compared to those with lower levels, as indicated by a Hazard Ratio (HR) of 1.84, with a 95% Confidence Interval (CI) of 1.17 to 2.89 (*p* < 0.05). The predictive accuracy of the model was substantial, as evidenced by the Area Under the Curve (AUC) for ROC curves at 3, 5, and 10 years, which were 0.873, 0.870, and 0.862, respectively. Furthermore, mediation analysis indicated that DM significantly influenced the relationship between the NLR and mortality, with a mediation effect of 6.57% (95% Confidence Interval [CI]: 0.64 to 15%; *p* = 0.02).

**Conclusion:**

Elevated NLR is significantly associated with an increased risk of all-cause mortality in patients with resolved HBV infection. Concurrently, DM acts as a partial mediator of this association.

## 1 Background

Hepatitis B virus (HBV) infection is recognized as a significant global health challenge (1, 2). The principal therapeutic endpoint in the management of chronic HBV infection is the seroclearance of hepatitis B surface antigen (HBsAg) from serum, a criterion that defines the achievement of a “functional cure.” However, even with the clearance of HBsAg, the complete eradication of HBV may not be possible due to the presence of covalently closed circular DNA (cccDNA) and the integration of HBV DNA into the host genome (3). Consequently, there is still a risk of HBsAg seroconversion, HBV reactivation, liver cirrhosis, and hepatocellular carcinoma (4). Resolved HBV infection denotes a stage characterized by viral elimination, marked by undetectable serum HBsAg and the presence of HBcAb seropositivity (4, 5). Despite affecting approximately 9.87 million US adults (6), this condition is often overlooked. Individuals with resolved HBV infection have higher mortality rates compared to those without chronic liver disease (7). A cohort study in the United States documented 741 fatalities over a span of 10.3 years, equating to a mortality rate of 21.4 percent (7). However, research on mortality and predictive factors for resolved HBV infection is scarce.

The NLR is an emerging biomarker for systemic inflammation, which could better reflect the state of systemic inflammation than a single indicator (8). Neutrophils play a critical role in the innate immune response. They secrete cytokines and chemokines that aid in the recruitment, activation, and coordination of other immune cells. In the pathogenesis of systemic inflammatory response syndrome, these secreted factors are essential for the function of dendritic cells, CD4+ T cells, and CD8+ T cells (9). Lymphocytes, pivotal for immune surveillance, are essential for combating external pathogens and monitoring cellular alterations (10). Fluctuations in lymphocyte counts are significant for assessing immune function and exert a critical regulatory influence on systemic inflammatory processes. The NLR indicates a balance between inflammatory and immune regulatory states and has shown significant prognostic value in diseases such as ulcerative colitis (11), liver failure (9), hemodialysis (12), and autoimmune encephalitis (13), highlighting its potential for further development and clinical application. Meanwhile, the relationship between diabetes and HBV infection remains a contentious issue. Previous research has identified HBV infection as a potential risk factor which could increase the risk of diabetes by 33 percent (14). However, some studies paradoxically suggest a protective effect of HBV against diabetes (15).

Despite a growing interest in the NLR as a prognostic indicator, there is a notable dearth of studies that establish its predictive value for patients with resolved hepatitis B infection. Furthermore, the intermediary role of diabetes remains to be elucidated. This study aims to investigate the link between the NLR score and all-cause mortality in resolved hepatitis B patients, as well as to explore the potential mediating effect of diabetes. The results will offer new assessment metrics for clinical evaluation, thereby aiding in the enhancement of clinical management for this patient cohort.

## 2 Methods

### 2.1 Study population

The NHANES, conducted by the Centers for Disease Control and Prevention, is a comprehensive cross-sectional study designed to assess the health and nutritional status of a sample representative of the U.S. population. This assessment encompasses interviews, physical examinations, dietary surveys, and laboratory tests. The study protocol has received formal approval from the National Center for Health Statistics Institutional Review Board, and all participants have provided informed consent.

**Figure 1:**
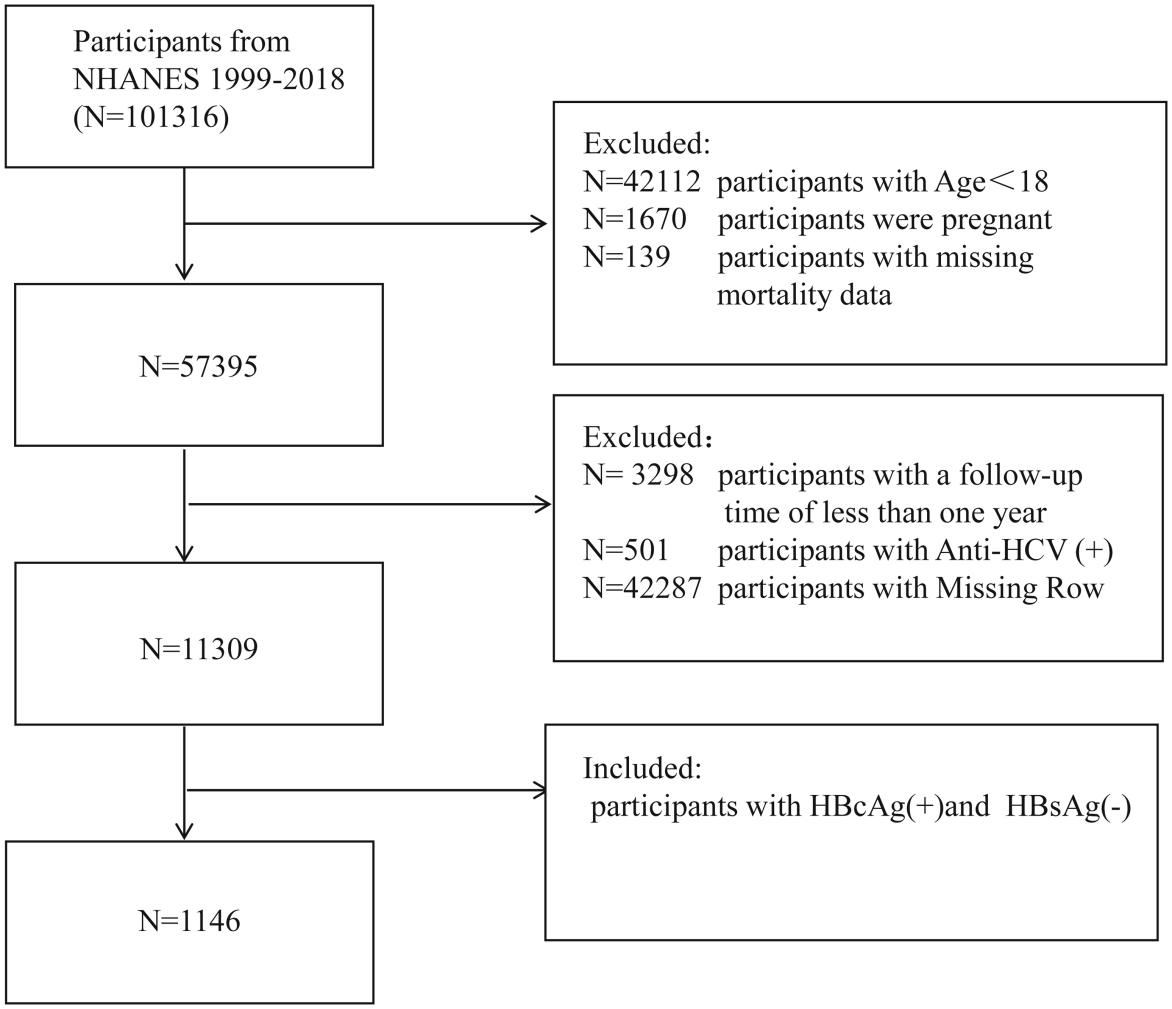
The flow chart of participants inclusion and exclusion in current study.

The research data were extracted from the NHANES program cycles conducted from 1999 to 2018. Participants were excluded based on the following criteria: (a) age under 18 years; (b) pregnancy; (c) do not meet the definition criteria for resolved HBV patients; (d) lost to follow-up; (e) individuals with less than 12 months of follow-up; (f) missing data for any covariates. Ultimately, 1,146 participants were included in the final analysis (Figure 1). Since this study does not involve exposure to personal identity information, it was approved by the National Center for Health Statistics Research Ethics Review Board, thus no additional ethical review was required.

### 2.2 Definition and measurement of resolved HBV infection and blood cell counts

Drawing on prior research, resolved HBV infection is characterized by the absence of detectable HBsAg and the presence of antibodies to hepatitis B core antigen (anti-HBc) (7). Details regarding the definition and laboratory procedures for anti-HBs positivity, as well as the criteria for eligible samples, are available in the NHANES Laboratory Methods Manual (6). The complete blood count is a routine blood test that provides a comprehensive health assessment and can reveal various conditions. The NLR is calculated by dividing the neutrophil count by the lymphocyte count (15).

### 2.3 All-cause mortality and other variables

The mortality data in this study was derived from the public-use linked mortality file released by NHANES in 2019. Deaths occurring before 1998 were categorized based on the ICD-9 classification system, while those from 1998 onwards were encoded according to the updated ICD-10 standards. The primary endpoint of this study was all-cause mortality, which includes death events from a variety of causes. This analysis utilized an anonymized dataset for secondary research. Data was collected from face-to-face interviews conducted by NHANES staff in participants’ homes, gathering demographic and health-related information, including age, gender, ethnicity, education level, marital status, and household income. At the mobile examination centers, participants’ height, weight, waist circumference, and body mass index (BMI) were measured. BMI is calculated as weight in kilograms divided by the square of height in meters, categorized as <18.5, 18.5–25, 25–30, and ≥30 kg/m^2^. Ethnicity was categorized as follows: Non-Hispanic Black(Black), Mexican American(Mexican), Non-Hispanic White(White), Other Hispanic(Hispanic), and Other Race-Including Multi-Racial(Other). Educational level was stratified into: college degree or above, high school or equivalent, and less than high school. Drinking history is categorized as present or absent. The FIB-4 score was calculated using the formula:FIB-4 = AST (IU/L) × age (years)/[PLT count (10^9^ /L) × √ ALT (IU/L)]. Smoking history was categorized into three groups: never (those who have smoked fewer than 100 cigarettes in their lifetime), Former (those who have smoked 100 or more cigarettes but are not currently smoking), and Current (those who have smoked 100 or more cigarettes and currently smoke on some days or daily). The Poverty Income Ratio (PIR) was calculated by dividing the total household income by the poverty line standard and was stratified as: ≤1.0, 1.0–3.0, and >3.0. Alanine transaminase (ALT), Aspartate aminotransferase (AST), Alkaline phosphatase (ALP), Gamma Glutamyl Transferase (GGT), Serum Creatinine (SCR), and Albumin are obtained from laboratory test results. DM is defined as: being told by a healthcare professional that you have diabetes; a glycated hemoglobin HbA1c of ≥6.5%, a fasting blood glucose of ≥7.0 mmoL/L, a random blood glucose of ≥11.1 mmoL/L, or a 2-h OGTT blood glucose of ≥11.1 mmoL/L; or the use of diabetes medication or insulin. Cardiovascular disease is defined as: ever being told you had coronary heart disease; ever being told you had angina/angina pectoris; ever being told you had a heart attack.

### 2.4 Statistical analysis

In this study, we leveraged NHANES examination weights to ensure our analysis accurately represented the U.S. civilian non-institutionalized population across survey cycles from 1999–2000 to 2017–2018, following the NHANES Analytic Guidelines^1^^,^ ^2^. Baseline characteristics were assessed using chi-square test or one-way ANOVA, with categorical data expressed as percentages and 95% CI, and continuous data as mean ± standard deviation. The ‘maxstat’ package was employed to determine the optimal NLR cutoff, distinguishing participants based on NLR levels. We explored the linear association between NLR and all-cause mortality in hypertensive patients using RCS models and evaluated the independent association between NLR and mortality in resolved HBV patients with weighted Cox proportional hazards models. Results were stratified into three models adjusting for various demographic and clinical parameters. Survival probabilities were assessed using the Kaplan–Meier method and log-rank tests, while the ‘timeROC’ package was used to evaluate NLR’s predictive accuracy for survival outcomes. Mediation analysis was conducted to investigate the indirect effects of DM on the relationship between NLR and all-cause mortality in resolved HBV patients. All statistical analyses were performed using R version 4.4 (R Foundation for Statistical Computing, Vienna, Austria). A two-tailed *p*-value of less than 0.05 was considered statistically significant.

## 3 Results

### 3.1 General characteristics

**Figure 2:**
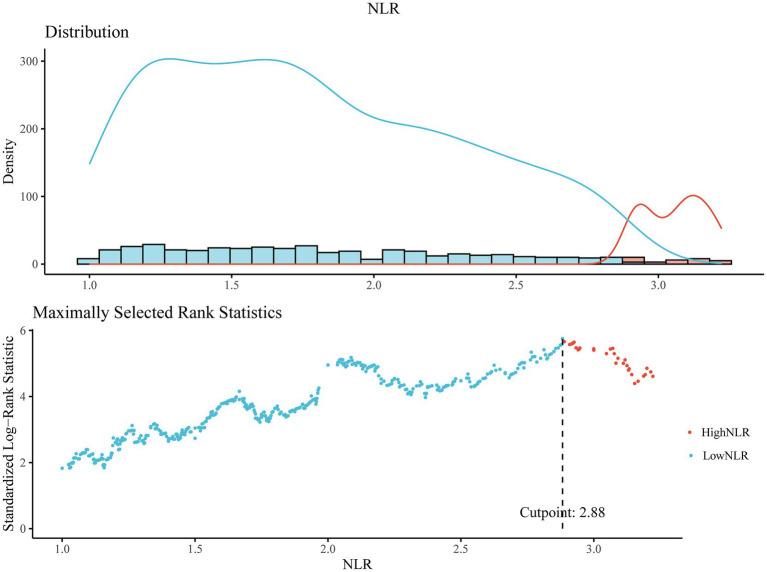
The cutoff point was calculated using the maximally selected rank statistics based on the ‘maxstat’ package and Standardized Log-Rank Statistic.

From 1999 to 2018, this study enrolled a total of 1,146 individuals with resolved HBV infection, with an average age of 54.30 ± 0.61 years. Males accounted for 57.92% (50.25, 65.59%) of the participants, while females accounted for 42.08% (35.37, 48.79%). As depicted in Figure 2, the optimal cutoff value for NLR was identified as 2.88, which divided the participants into a high NLR group (NLR > 2.88, *n* = 172) and a low NLR group (NLR ≤ 2.88, *n* = 974) (Figure 2). Participants in the high NLR group had a higher proportion of whites and exhibited higher white blood cell and neutrophil counts (*p* < 0.0001), as well as lower lymphocyte counts (*p* < 0.0001) compared to the low NLR group. Further characteristics of the participants are detailed in Table 1.

**Table 1:** Characteristic of participants.

Variable	Total (*n* = 1,146)	High NLR (*n* = 172)	Low NLR (*n* = 974)	*p-*value
Age	54.30 ± 0.61	56.70 ± 2.25	53.86 ± 0.60	0.22
PIR	2.54 ± 0.10	2.50 ± 0.18	2.54 ± 0.11	0.81
BMI	27.75 ± 0.24	27.96 ± 0.49	27.71 ± 0.26	0.64
FIB.4	1.26 ± 0.03	1.28 ± 0.09	1.26 ± 0.03	0.86
WBC	7.03 ± 0.11	8.13 ± 0.21	6.83 ± 0.11	<0.0001
LYM	2.18 ± 0.04	1.53 ± 0.04	2.30 ± 0.05	<0.0001
NEU	4.02 ± 0.08	5.73 ± 0.17	3.71 ± 0.07	<0.0001
Follow up time	103.54 ± 4.90	98.97 ± 8.85	104.38 ± 5.21	0.56
ALT	25.15 ± 0.57	23.33 ± 1.27	25.49 ± 0.63	0.13
AST	25.27 ± 0.53	23.05 ± 0.94	25.67 ± 0.58	0.01
ALP	71.77 ± 1.19	76.61 ± 3.34	70.88 ± 1.22	0.11
GGT	31.93 ± 1.56	30.64 ± 2.41	32.16 ± 1.84	0.63
SCR	0.91 ± 0.02	0.99 ± 0.05	0.89 ± 0.02	0.07
Albumin	42.90 ± 0.17	42.74 ± 0.37	42.93 ± 0.16	0.59
NLR	2.03 ± 0.05	3.84 ± 0.09	1.69 ± 0.03	<0.0001
Year				0.5
1999–2000	18.34 (13.38,23.30)	19.51 (10.97,28.04)	18.13 (13.58,22.67)	
2003–2004	22.23 (16.31,28.16)	27.54 (14.58,40.50)	21.26 (16.34,26.18)	
2013–2014	21.12 (16.52,25.71)	14.54(6.94,22.15)	22.32 (17.82,26.82)	
2015–2016	21.57 (18.42,24.72)	20.29 (12.26,28.32)	21.80 (18.39,25.22)	
2017–2018	16.74 (11.11,22.36)	18.12(7.26,28.98)	16.49 (11.42,21.55)	
Sex				0.04
Female	42.08 (35.37,48.79)	30.69 (20.26,41.11)	44.17 (39.06,49.28)	
Male	57.92 (50.25,65.59)	69.31 (58.89,79.74)	55.83 (50.72,60.94)	
Race				<0.0001
White	42.22 (34.52,49.93)	59.48 (50.60,68.36)	39.06 (33.85,44.28)	
Black	24.06 (20.20,27.92)	16.94 (11.79,22.09)	25.36 (21.44,29.28)	
Mexican	3.29(2.19, 4.38)	3.20 (1.22,5.17)	3.30 (2.10,4.51)	
Hispanic	8.55(5.15,11.95)	4.76 (0.85, 8.67)	9.24 (5.89,12.59)	
Other	21.89 (17.14,26.63)	15.62(9.22,22.02)	23.03 (18.85,27.21)	
Education level				0.06
Less than high school	11.96(9.46,14.47)	9.05 (5.33,12.76)	12.50 (9.91,15.09)	
High school or equivalent	36.30 (30.49,42.11)	46.17 (35.78,56.56)	34.49 (29.77,39.22)	
College or above	51.74 (43.40,60.08)	44.79 (34.04,55.53)	53.01 (47.26,58.77)	
Smoke				0.14
Never	50.28 (43.48,57.08)	41.87 (33.24,50.49)	51.82 (47.88,55.76)	
Former	24.83 (20.96,28.70)	27.85 (20.22,35.48)	24.28 (20.47,28.09)	
Now	24.88 (19.45,30.32)	30.28 (20.58,39.98)	23.90 (19.56,28.23)	
DM				0.03
No	81.59 (71.72,91.46)	72.31 (62.64,81.98)	83.29 (80.31,86.27)	
Yes	18.41 (15.44,21.38)	27.69 (18.02,37.36)	16.71 (13.73,19.69)	
Mortality				<0.0001
Assumed alive	84.20 (73.63,94.78)	67.26 (58.88,75.64)	87.31 (84.31,90.30)	
Assumed deceased	15.80 (13.20,18.39)	32.74 (24.36,41.12)	12.69(9.70,15.69)	
CVD				0.01
No	91.91 (80.91,102.92)	86.41 (81.01,91.81)	92.92 (90.89,94.96)	
Yes	8.09(6.17, 10.00)	13.59 (8.19,18.99)	7.08 (5.04, 9.11)	
PIR group				0.06
≤1.0	20.22 (16.56,23.88)	15.69 (10.28,21.10)	21.05 (17.06,25.04)	
1.0–3.0	43.66 (36.65,50.68)	54.82 (44.90,64.73)	41.62 (36.47,46.78)	
>3	36.11 (29.07,43.16)	29.49 (19.48,39.50)	37.33 (31.15,43.50)	
BMI group				0.62
<18.5	1.71(0.69, 2.73)	1.76(−0.61,4.13)	1.70(0.56,2.84)	
18.5–25	33.58 (27.98,39.17)	35.21 (25.99,44.43)	33.28 (28.75,37.80)	
25–30	35.61 (29.05,42.16)	30.09 (19.77,40.40)	36.62 (31.39,41.85)	
≥30	29.11 (24.14,34.08)	32.95 (23.53,42.36)	28.41 (24.54,32.27)	
Alcohol history				0.57
no	16.98 (13.46,20.50)	14.91(7.58,22.23)	17.36 (14.16,20.56)	
yes	83.02 (73.46,92.58)	85.09 (77.77,92.42)	82.64 (79.44,85.84)	

PIR, poverty income ratio; WBC, white blood cell; LYM, lymphocyte; NEU, neutrophil; NLR, neutrophil-to-lymphocyte ratio; BMI, body mass index; ALT, alanine aminotransferase; AST, aspartate aminotransferase; ALP, alkaline phosphatase; GGT, gamma glutamyl transferase; FIB-4, fibrosis-4 index; SCR, serum creatinine; DM, diabetes mellitus; CVD, cardiovascular disease.

### 3.2 Association between NLR and all-cause mortality

During an average follow-up duration of 103.54 ± 4.90 months, 207 out of 1,146 individuals with resolved HBV infection succumbed to death, representing about 18.06% of the cohort. The high NLR group exhibited a higher mortality rate compared to the low NLR group (Table 1). As demonstrated in Table 2, the unadjusted model revealed that an increase in the NLR was significantly associated with a substantial rise in the risk of all-cause mortality (HR = 2.67, 95% CI: 1.86–3.84, *p* < 0.0001). Table 3 further illustrates that, after multivariable adjustment, each unit increase in NLR was associated with an 86% increase in the risk of all-cause mortality in Model 1 (HR = 1.86, 95% CI: 1.15–3.00, *p* < 0.0001), a 77% increase in Model 2 (HR = 1.77, 95% CI: 1.05–2.97, *p* < 0.05), and a 72% increase in Model 3 (HR = 1.72, 95% CI: 1.04–2.86, *p* < 0.05). Considering that the resolved HBV infection cohort in this study is predominantly composed of the white population, we specifically adjusted for ethnicity to eliminate potential biases in the study outcomes. As shown in Supplementary Table S1, after excluding the white population, a significant association between high NLR and the risk of all-cause mortality in resolved HBV patients among other ethnic groups still persists. Cox regression analysis demonstrated that the risk for all-cause mortality significantly increased in the higher-NLR group from the crude model (HR 2.53, 95% CI 1.63–3.94, *p* < 0.0001) to the adjusted models (Models 1, 2, and 3) (HR 2.08, 95% CI 1.22–3.56, *p* = 0.01; HR 1.76, 95% CI 1.10–2.82, *p* = 0.02; HR 1.84, 95% CI 1.17–2.89, *p* = 0.01). Similarly, as shown in Supplementary Table S2, after excluding the impact of trauma/accidents and performing multivariable model adjustments, we ultimately found that the results were consistent with the trends of our aforementioned findings.

**Table 2:** Subgroup analyses of NLR and mortality risk in participants.

Character	Low NLR	High NLR	*p*	*p* for interaction
Sex				0.172
Female	Ref	1.827 (0.838,3.983)	0.13	
Male	Ref	3.341 (2.013,5.546)	<0.0001	
Race				0.049
White	Ref	2.418 (1.307,4.471)	0.005	
Black	Ref	1.496 (0.794,2.820)	0.213	
Mexican	Ref	1.151 (0.252,5.247)	0.856	
Hispanic	Ref	19.807 (7.340,53.451)	<0.0001	
Other	Ref	3.066 (0.809,11.621)	0.099	
Education level				0.12
Less than high school	Ref	6.097 (2.937,12.657)	<0.0001	
High school or equivalent	Ref	3.360 (1.982,5.697)	<0.0001	
College or above	Ref	1.803 (0.783,4.149)	0.166	
Smoking status				0.794
Never	Ref	2.694 (1.353,5.364)	0.005	
Former	Ref	2.485 (1.448,4.262)	<0.001	
Now	Ref	3.085 (1.495,6.365)	0.002	
Alcohol history				0.514
No	Ref	1.837 (0.522,6.460)	0.343	
Yes	Ref	2.874 (1.832,4.507)	<0.0001	
CVD				0.469
Yes	Ref	1.759 (0.792,3.909)	0.165	
No	Ref	2.739 (1.772,4.234)	<0.0001	
DM				0.171
No	Ref	3.054 (1.932,4.826)	<0.0001	
Yes	Ref	1.652 (0.778,3.508)	0.191	
BMI				0.627
18.5–25	Ref	2.168 (0.880,5.339)	0.092	
<18.5	Ref	1.738 (0.466,6.480)	0.41	
25–30	Ref	3.507 (2.091,5.880)	<0.0001	
≥30	Ref	2.601 (1.275,5.303)	0.009	
PIR				0.583
≤1.0	Ref	2.399 (1.230,4.679)	0.01	
1.0–3.0	Ref	2.308 (1.286,4.143)	0.005	
>3	Ref	3.311 (1.673,6.551)	<0.001	

HR, hazard ratio; 95%CI, confidence interval; Ref, reference; CVD, Cardiovascular disease; DM, diabetes mellitus; BMI, Body Mass Index; PIR, poverty income ratio.

**Table 3:** The relationships between NLR and mortality in participants with resolved HBV.

	Crude model		Model 1		Model 2		Model 3	
Character	95%CI	*p*	95%CI	*p*	95%CI	*p*	95%CI	*p*
Low NLR	Ref		Ref		Ref		Ref	
High NLR	2.67 (1.86,3.84)	<0.0001	1.86 (1.15,3.00)	0.01	1.77 (1.05, 2.97)	0.03	1.72 (1.04, 2.86)	0.04

Crude model, unadjusted; Model 1, adjusted for age, ethnicity, BMI, smoke, alcohol; Model 2 adjusted for age, ethnicity, BMI, smoke, alcohol, FIB-4, AST/ALT, ALP, GGT, Albumin; Model3 adjusted for age, ethnicity, BMI, smoke, alcohol, FIB-4, AST/ALT, ALP, GGT, Albumin, SCR, CVD.

**Figure 3:**
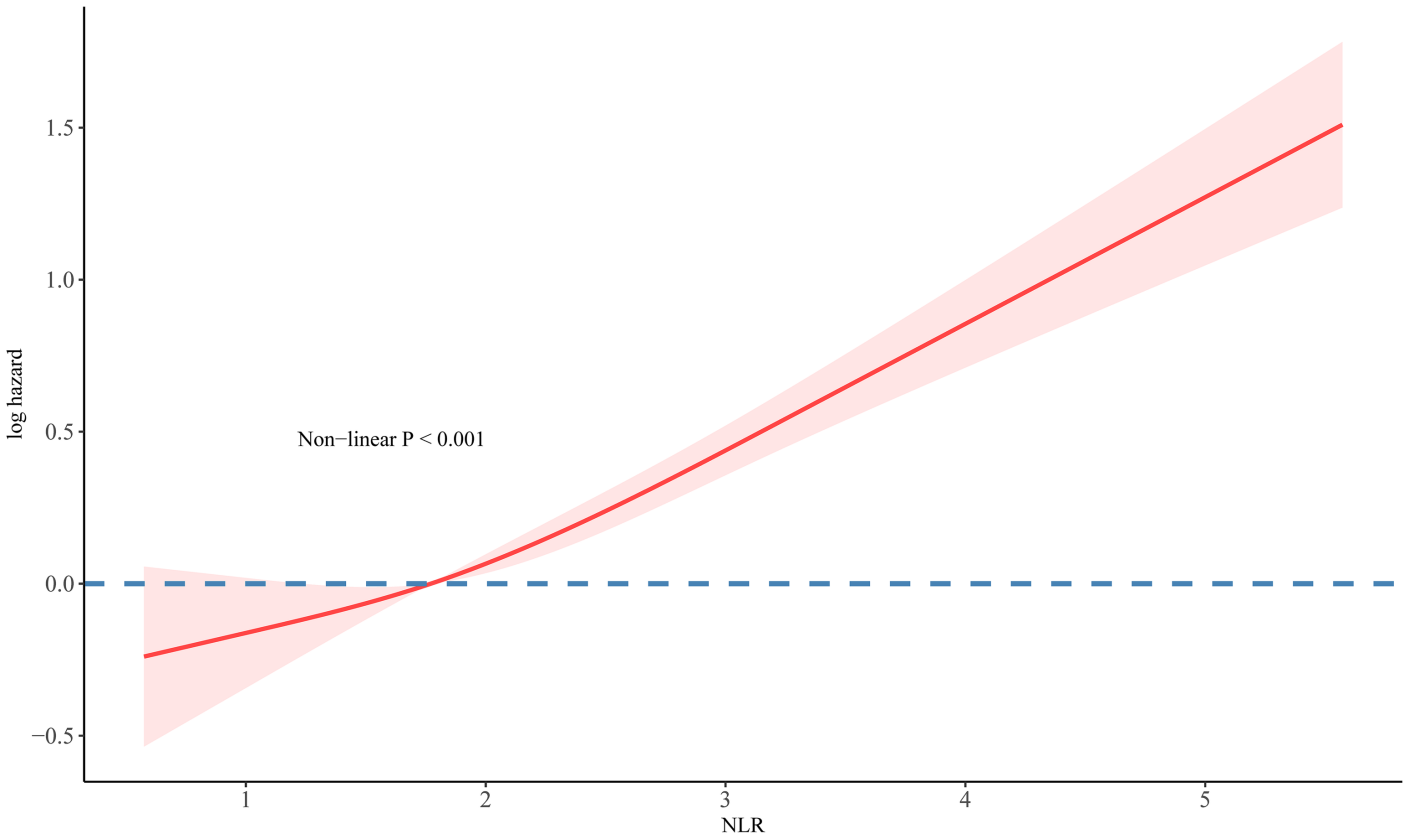
The association of NLR with all-cause mortality in participants with resolved HBV visualized by RCS.

**Figure 4:**
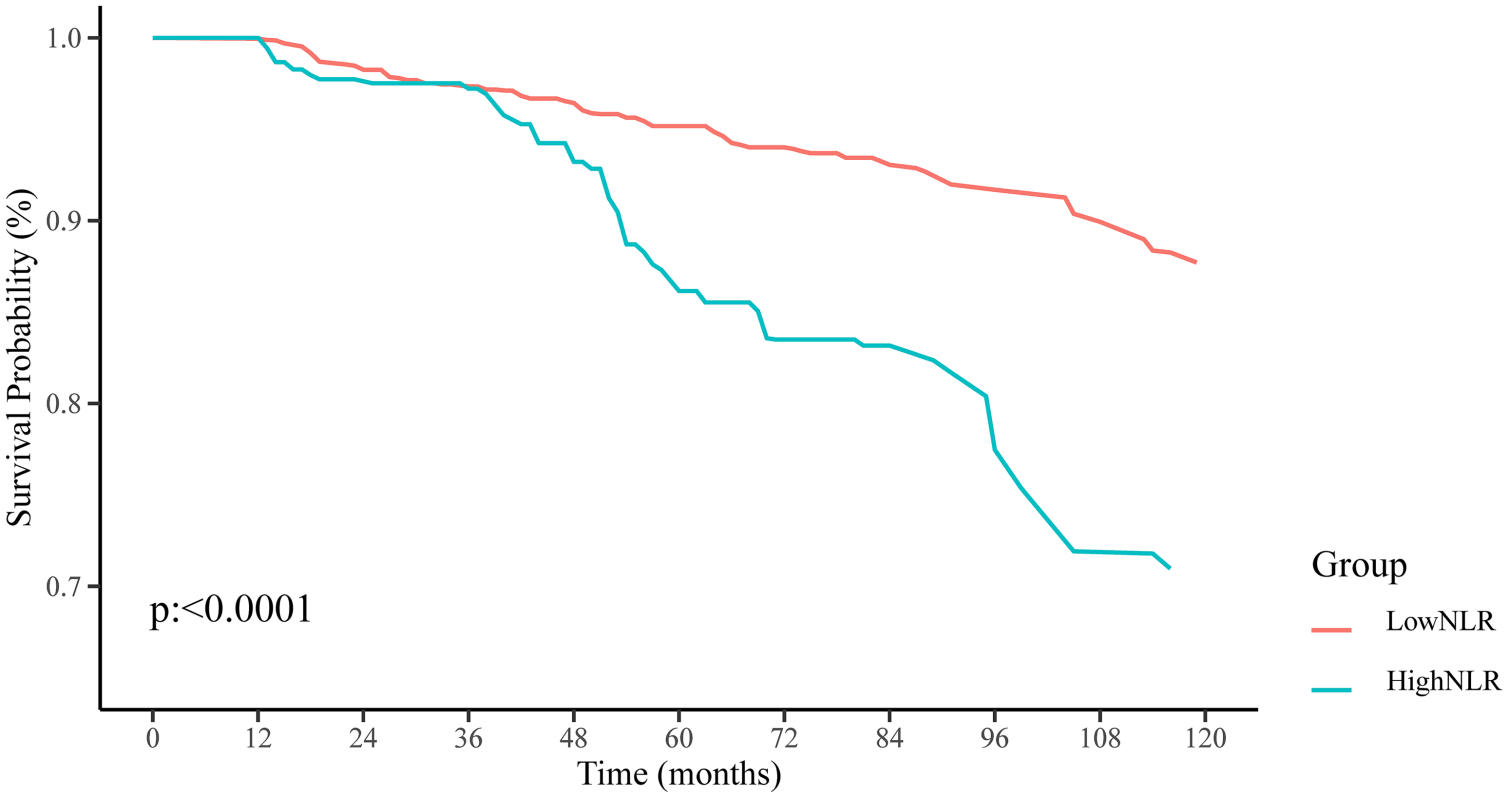
Kaplan–Meier survival curves and at-risk population proportions (%) for resolved HBV infected patients with higher (>2.88) and lower (≤2.88) NLR value.

The use of RCS analysis confirmed a positive linear correlation between the NLR and the risk of all-cause mortality in patients with resolved HBV infection (non-linear *p* < 0.001), as shown in Figure 3. Additionally, Kaplan–Meier survival curves indicated a significant difference in all-cause mortality rates between the high and low NLR groups (*p* < 0.0001), with the high NLR group exhibiting a lower survival rate (Figure 4).

### 3.3 ROC analysis for NLR in resolved HBV mortality prediction

**Figure 5:**
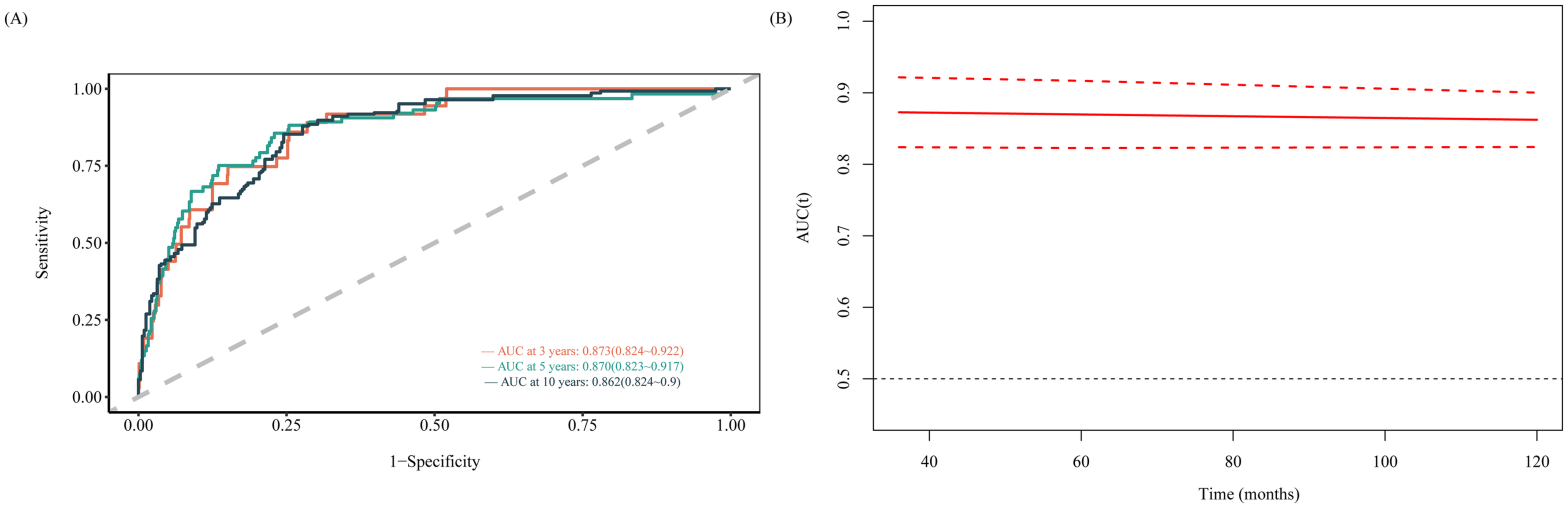
Time-dependent ROC curves and time-dependent AUC values (with 95% confidence band) of the NLR for predicting all-cause mortality (**A** sensitivity and **B** AUC).

Time-dependent ROC analysis was used to evaluate the predictive power of NLR for all-cause mortality in individuals with resolved HBV infection. The analysis revealed that the AUC for NLR in predicting the risk of all-cause mortality at 3, 5, and 10 years was 0.873 (95% CI: 0.824–0.922), 0.870 (95% CI: 0.823–0.917), and 0.862 (95% CI: 0.824–0.900), respectively (Figure 5). These findings indicate that NLR serves as a significant predictor of all-cause mortality risk, demonstrating both short-term and long-term effectiveness in the resolved HBV infection population.

### 3.4 Mediating effect of diabetes on NLR and all-cause mortality in resolved HBV patients

**Figure 6:**
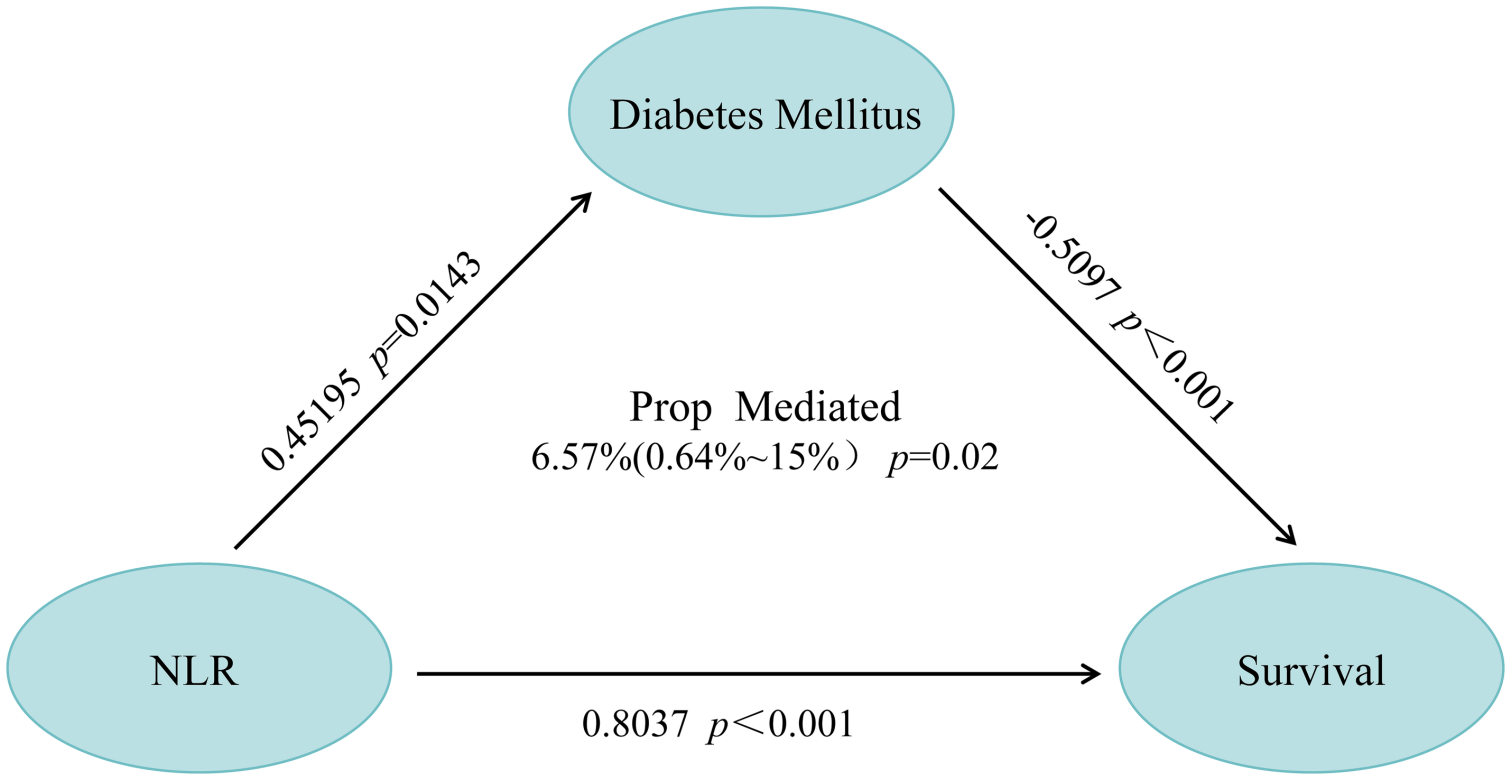
Mediation path diagram: the impact of DM on the association between NLR ratio changes and survival in resolved HBV infected patients.

Excluding mediator variables, the direct effect of NLR on all-cause mortality in individuals with resolved HBV infection is significant (Table 3). Furthermore, when DM is included as a mediating variable in the model, a significant mediating effect of DM on the relationship between NLR and all-cause mortality in resolved HBV patients is observed (*β* = −0.5097, *p* < 0.001). Additionally, NLR significantly influences the presence of diabetes (*β* = 0.45195, *p* = 0.0143). After conducting proportional mediation analysis, it is found that DM accounts for 6.57% (0.64–15%) of the all-cause mortality in resolved HBV patients (*p* = 0.02, Figure 6). This proportion indicates that DM plays a partial mediating role in the relationship between NLR and all-cause mortality in individuals with resolved HBV infection. However, as Supplementary Figure S1 shows, our subgroup analysis results indicate that the mediating effect of DM in this study is statistically significant in the white group, while the mediating effect in other ethnic groups is not statistically significant.

## 4 Discussion

In the context of HBV infection treatment and prognosis assessment, the population with resolved HBV infection appears to be overlooked. Previous reports have highlighted the occurrence of *de novo* hepatitis caused by resolved HBV infection in liver transplant recipients (16–19). Crespo et al. (20) described a case of acute liver failure in a patient with resolved HBV infection following liver transplantation. Current research primarily focuses on the risk of HBV reactivation in patients undergoing immunosuppressive therapy (7, 21–23). Huang et al. (7) demonstrated that the presence of anti-HBs antibodies is associated with a reduced risk of HBV reactivation. Patients with resolved HBV infection who experience reactivation are at risk of severe liver complications, including liver failure and death (21, 24, 25). Consequently, this underscores the necessity for enhanced public health surveillance and the development of additional risk prediction models and prognostic indicators for patients with resolved HBV infection.

In chronic HBV infection, neutrophil and lymphocyte dynamics are intricately linked to viral immune modulation and hepatic pathology. Neutrophilia correlates with systemic inflammation, whereas lymphopenia signifies compromised cellular immunity (25, 26). An elevated NLR, indicative of systemic inflammation, is associated with enhanced hepatic inflammation and fibrosis progression in HBV infection (27, 28). In resolved HBV patients, NLR may predict liver inflammation and extrahepatic inflammatory complications, potentially impacting long-term survival. Elevated NLR reflects persistent inflammation and immune dysregulation, which could be linked to covert liver disease progression, metabolic syndrome, and increased cardiovascular risk, thereby influencing all-cause mortality (29–31). Our study finds that NLR correlates with all-cause mortality in patients with resolved HBV infection. Elevated NLR is significantly linked to resolved HBV and independently predicts patient survival. This association remains significant even after adjusting for common risk factors, highlighting NLR’s potential as a biomarker for patient management and clinical outcomes.

In a large cohort study based on population data from Taiwan and Hong Kong, DM was identified as one of the independent risk factors for disease progression in patients with chronic hepatitis B (CHB) (32). Another study reveals that patients with CHB who also have diabetes are at an elevated risk of all-cause mortality compared to those without diabetes, suggesting a potential link between diabetes and the progression of hepatitis B (33). This association may be mediated through multiple mechanisms. Hyperglycemia and insulin resistance, which are hallmark features of DM, are known to exacerbate liver damage by promoting inflammation, lipotoxicity, and oxidative stress pathways (34, 35). Moreover, in patients with CHB, the presence of DCHBM may intensify liver inflammation and fibrosis (36). Regarding potential mechanisms, DM may lead to metabolic disorders in the liver, thereby affecting the liver’s immune control of HBV, which could promote viral replication and the progression of liver disease (37). Additionally, the characteristics of metabolic syndrome in patients with DM, such as hypertension and dyslipidemia, may also be associated with an increased risk of all-cause mortality in patients with CHB (38). However, these viewpoints require further research evidence to be proven. Our research indicates that DM partially mediates the impact of NLR on all-cause mortality in the resolved HBV infection population, and diabetes is also a risk factor for all-cause mortality in this group. Based on these results, we infer that improving diabetes status is likely to help reduce the mortality risk in this population. Therefore, we recommend that clinicians incorporate diabetes-related indicators, such as blood glucose levels, into the follow-up metrics for this group.

This study carries certain limitations. Firstly, the retrospective nature of the study may introduce selection bias. Furthermore, the inherent constraints of the NHANES database result in an absence of detailed records concerning the collection phase of neutrophil and lymphocyte data, preventing the determination of the specific stage within the infection cycle at which these data were collected in patients with resolved hepatitis B. Additionally, the database lacks HBV DNA results and data on patients with resolved hepatitis B from the periods 2000–2003 and 2004–2013. These limitations may further affect the generalizability of the results. We accounted for multiple covariates, but unknown confounding factors may still impact the precision of our conclusions. Nonetheless, this study provides valuable references for research in the relevant field. Therefore, it would be beneficial for future studies to employ prospective methods to further explore the relationship between the NLR and mortality rates in patients with resolved HBV infection. This approach would help to confirm the efficacy of NLR as a predictive marker for resolved hepatitis B and solidify the scientific basis for clinical diagnosis and treatment protocols.

## 5 Conclusion

In summary, our analysis of 1,146 resolved HBV patients from the NHANES database (1999–2018) revealed an association between NLR and the risk of all-cause mortality in these patients during long-term follow-up. Our findings underscore the importance of incorporating NLR into routine clinical practice as a biomarker for predicting all-cause mortality in resolved HBV patients. Additionally, our results suggest that DM plays a partial mediating role in the relationship between NLR and all-cause mortality in resolved HBV patients. This indicates that resolved HBV patients with comorbid DM should receive increased attention in clinical practice.

## Data Availability

Publicly available datasets were analyzed in this study. The National Health and Nutrition Examination Survey (NHANES) data are publicly available at https://www.cdc.gov/nchs/nhanes/index.htm.

## Footnotes

^1^ https://wwwn.cdc.gov/nchs/nhanes/tutorials/weighting.aspx

^2^ https://websites.umich.edu/~surveymethod/asda/
