# Supplementary material for: The neutrophil–lymphocyte ratio as a risk factor for all-cause mortality among individuals with resolved HBV infection: evidence from the NHANES 1999–2018
Source: Front Public Health. 2025 Jan 15;12:1493439. doi: 10.3389/fpubh.2024.1493439 (PMC11775152; doi:10.3389/fpubh.2024.1493439)
Supplement: Supplementary file 1 [file Data_Sheet_1.PDF]

Supplementary Table S1 The relationships between NLR and mortality in participants with resolved HBV: a multivariable regression analysis excluding the non-hispanic white

Supplementary Table S2 The relationships between NLR and mortality in participants with resolved HBV: a multivariable regression analysis with trauma/accident-related deaths excluded

Supplementary FigureS1 The impact of diabetes mellitus on the association between neutrophil-to-lymphocyte ratio changes and survival in resolved hepatitis B virus infected patients: a comparative analysis across different ethnicities

Supplementary TableS1 The relationships between NLR and mortality in participants with resolved HBV: a multivariable regression analysis excluding the non-hispanic white

	crude model		Model 1		Model 2		Model 3	
Character	95%CI	*p*	95%CI	*p*	95%CI	*p*	95%CI	*p*
lowNLR	Ref		Ref		Ref		Ref	
highNLR	2.53(1.63,3.94)	<0.0001	2.08(1.22, 3.56)	0.01	1.76(1.10, 2.82)	0.02	1.84(1.17, 2.89)	0.01

Note:Crude Model, unadjusted;Model 1, adjusted for age, ethnicity, BMI, smoke, alcohol;Model 2 adjusted for age, ethnicity, BMI, smoke, alcohol, FIB-4, AST/ALT, ALP, GGT, Albumin;Model 3 adjusted for age, ethnicity, BMI, smoke, alcohol, FIB-4, AST/ALT, ALP, GGT, Albumin, SCR, CVD.

Supplementary TableS2 The relationships between NLR and mortality in participants with resolved HBV: a multivariable regression analysis with trauma/accident-related deaths excluded

	crude model		Model 1		Model 2		Model 3	
Character	95%CI	*p*	95%CI	*p*	95%CI	*p*	95%CI	*p*
lowNLR	Ref		Ref		Ref		Ref	
highNLR	2.71(1.88,3.91)	<0.0001	1.87(1.15, 3.03)	0.01	1.78(1.06, 3.00)	0.03	1.74(1.05, 2.88)	0.03

Note:Crude Model, unadjusted;Model 1, adjusted for age, ethnicity, BMI, smoke, alcohol;Model 2 adjusted for age, ethnicity, BMI, smoke, alcohol, FIB-4, AST/ALT, ALP, GGT, Albumin;Model 3 adjusted for age, ethnicity, BMI, smoke, alcohol, FIB-4, AST/ALT, ALP, GGT, Albumin, SCR, CVD

Supplementary FigureS1 can be found in the uploaded image.
