# Supplementary figures and images for: The neutrophil–lymphocyte ratio as a risk factor for all-cause mortality among individuals with resolved HBV infection: evidence from the NHANES 1999–2018
Source: Front Public Health. 2025 Jan 15;12:1493439. doi: 10.3389/fpubh.2024.1493439 (PMC11775152; doi:10.3389/fpubh.2024.1493439)

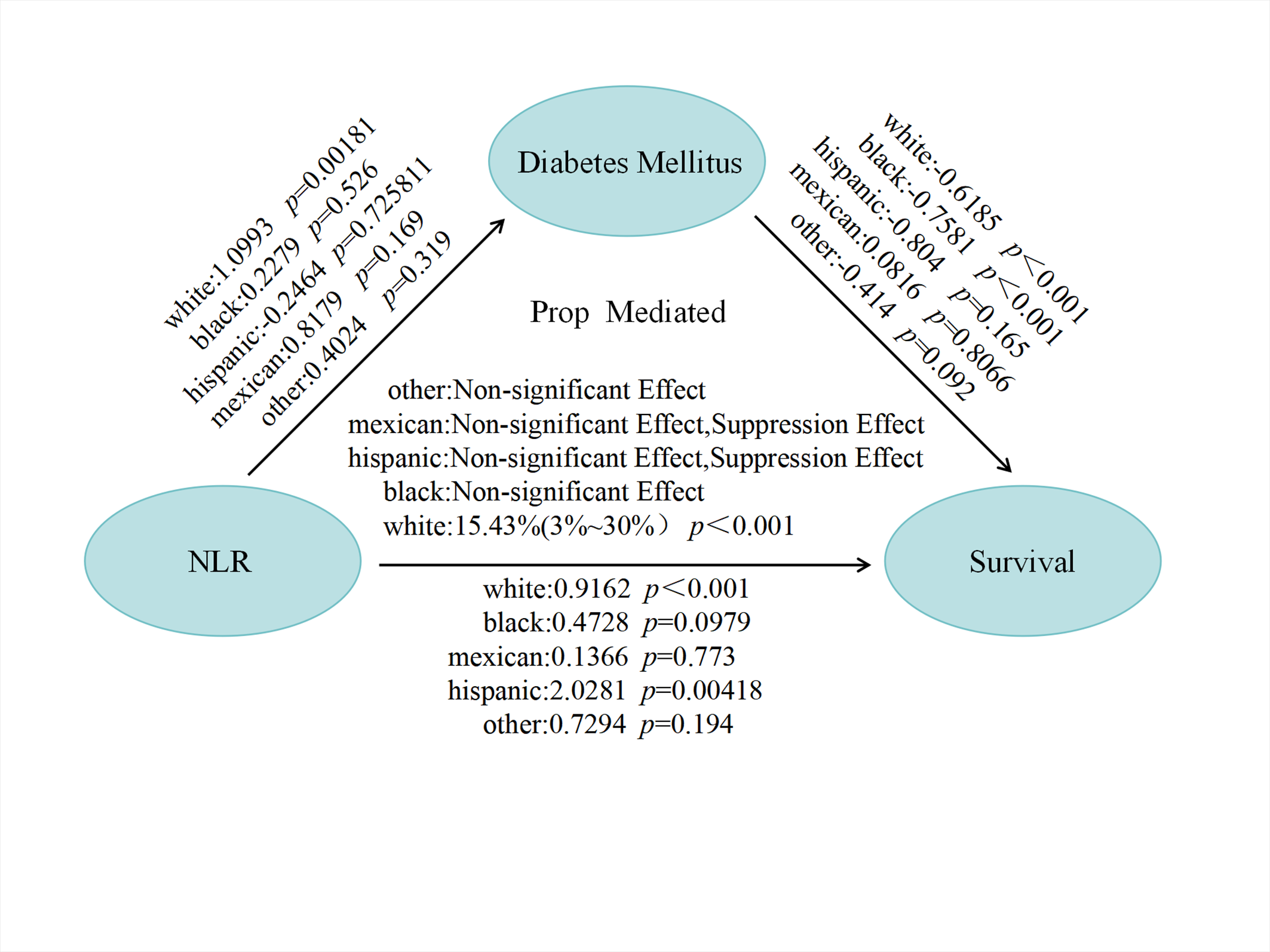

Supplement: Supplementary file 2 [file Image_1.TIF]
